# Dementia imaging in clinical practice: a European-wide survey of 193 centres and conclusions by the ESNR working group

**DOI:** 10.1007/s00234-019-02188-y

**Published:** 2019-03-09

**Authors:** M. W. Vernooij, F. B. Pizzini, R. Schmidt, M. Smits, T. A. Yousry, N. Bargallo, G. B. Frisoni, S. Haller, F. Barkhof

**Affiliations:** 1 Department of Radiology & Nuclear Medicine, Erasmus MC University Medical Center, Rotterdam, The Netherlands; 2 Department of Epidemiology, Erasmus MC University Medical Center, Rotterdam, The Netherlands; 3 Neuroradiology, Department of Diagnostics and Pathology, Verona University Hospital, Verona, Italy; 4 Department of Neurology, Clinical Division of Neurogeriatrics, Medical University Graz, Graz, Austria; 5 Lysholm Department of Neuroradiology, UCL Institute of Neurology, London, UK; 6 Magnetic Resonance Image Core Facility, IDIBAPS and Center of Diagnostic Image (CDIC), Hospital Clinic, Barcelona, Spain; 7 University Hospitals and University of Geneva, Geneva, Switzerland; 8 CIRD - Centre d’Imagerie Rive Droite|, Geneva, Switzerland; 9 Department of Surgical Sciences, Radiology, Uppsala University, Uppsala, Sweden; 10 Faculty of Medicine, University of Geneva, Geneva, Switzerland; 11 Department of Radiology and Nuclear Medicine, VU University Medical Centre, Amsterdam UMC, Amsterdam, The Netherlands

**Keywords:** Imaging, Dementia, MRI, Survey

## Abstract

**Purpose:**

Through a European-wide survey, we assessed the current clinical practice of imaging in the primary evaluation of dementia, with respect to standardised imaging, evaluation and reporting.

**Methods:**

An online questionnaire was emailed to all European Society of Neuroradiology (ESNR) members (*n* = 1662) and non-members who had expressed their interest in ESNR activities in the past (*n* = 6400). The questionnaire featured 42 individual items, divided into multiple choice, single best choice and free text answers. Information was gathered on the context of the practices, available and preferred imaging modalities, applied imaging protocols and standards for interpretation, reporting and communication.

**Results:**

A total of 193 unique (non-duplicate) entries from the European academic and non-academic institutions were received from a total of 28 countries. Of these, 75% were neuroradiologists, 12% general radiologists and 11% (neuro) radiologists in training. Of responding centres, 38% performed more than five scans/week for suspected dementia. MRI was primarily used in 72% of centres. Over 90% of centres acquired a combination of T2w, FLAIR, T1w, DWI and T2*w sequences. Visual rating scales were used in 75% of centres, most often the Fazekas and medial temporal atrophy scale; 32% of respondents lacked full confidence in their use. Only 23% of centres performed volumetric analysis. A minority of centres (28%) used structured reports.

**Conclusions:**

Current practice in dementia imaging is fairly homogeneous across Europe, in terms of image acquisition and image interpretation. Hurdles identified include training on the use of visual rating scales, implementation of volumetric assessment and structured reporting.

**Electronic supplementary material:**

The online version of this article (10.1007/s00234-019-02188-y) contains supplementary material, which is available to authorized users.

## Introduction

Worldwide populations are ageing, with an accompanying increase in prevalence of cognitive problems and dementia. At present, 50 million people worldwide are living with dementia, of whom the majority with Alzheimer’s disease as its most common subtype, and this number is predicted to double every 20 years to reach over 130 million by 2050 [1].

Nowadays, brain imaging plays a key role in the diagnosis and evaluation of patients suspected of dementia. Structural imaging, either with computed tomography (CT) or magnetic resonance imaging (MRI), is recommended at least once in the diagnostic workup of patients with cognitive impairment [2]. Whereas these exams initially served the goal to exclude alternative causes of dementia than neurodegeneration, which are potentially treatable (e.g. subdural hematomas or brain tumours), progressive insight has led to a role of imaging markers to help establish a positive diagnosis of dementia subtype. The latter is of increasing importance in light of prognosis, disease-modifying therapies and emerging treatment options. In particular, patterns of cerebral atrophy (including hippocampal atrophy) and presence and burden of vascular lesions can point towards a specific underlying diagnosis [3, 4]. This is reflected by the inclusion of neuroimaging markers into the diagnostic and research criteria for Alzheimer’s disease [5–7] and for vascular dementia [8, 9].

As a result of the above, radiologists both in academic and in non-academic centres are increasingly confronted with scans from patients evaluated at memory clinics. Yet, guidelines or recommendations on how to best manage the imaging, interpretation and reporting in this patient group are virtually non-existent [2]. Some clinical textbooks have published a proposed minimum set of MR imaging criteria for the evaluation of memory clinic patients [3, 4], consisting of 3D T1-weighted imaging, fluid-attenuated inversion recovery (FLAIR), turbo-spin or fast-spin T2-weighted images, diffusion-weighted images (DWI) and T2*-weighted gradient-recalled echo (GRE) imaging. Yet, to what extent such a suggested protocol is feasible and actually applied in clinical settings is largely unknown. For the interpretation of patterns of atrophy and vascular burden, several semi-quantitative visual rating scales have been reported [10–13] and validated [14, 15]. Again, whether these are actually used in everyday clinical practice across Europe also remains uncertain.

Apart from conventional structural neuroimaging, there is a large body of recent research into the use of advanced imaging markers derived from perfusion imaging (e.g. arterial spin labelling (ASL)), functional imaging (fMRI) or tissue microstructure (diffusion tensor imaging) for early detection of dementia or those at risk to develop to disease [16]. Also, developments are made to use quantitative information such as volumetric measures as potential diagnostic markers [17]. If such markers are expected to be implemented into clinical practice, it is useful to gauge the acceptance within the clinical community and potential hurdles associated with implementation.

In a recent survey conducted among memory clinics in the Netherlands, 75% of respondents reported that no standardised assessment of acquired images was performed [18]. This is in contradiction with the growing role of imaging in subtyping dementia, assessing diagnosis earlier and the inclusion of patients in clinical trials. The European Society of Neuroradiology (ESNR) therefore sought to assess the current clinical practice of imaging in the primary evaluation of dementia and determine potential hurdles to act upon. To this end, a working group was established which mapped the current landscape using a pan-European survey distributed among ESNR members and affiliates, addressing the context of the practices available and preferred imaging modalities, applied imaging protocols (including use of advanced imaging) and standards for interpretation, reporting and communication. The results of this survey as well as conclusions by the working group are reported in this manuscript.

## European survey on dementia imaging

### Methods

An online questionnaire was designed using Google forms open-access toolbox (Google.com, Mountain View, CA, USA). Questions were assembled by the members of the ESNR Working Group (authors MWV, FBP, FB, SH) and further modified/added by the ESNR Diagnostic Committee (MWV, TY, MS, NB) as well as by 2 members of the European Association of Neurology (EAN) committee (GF and RS, the latter acting as co-chair of the Panel of Dementia and Cognitive Disorders of the EAN). The questionnaire featured 42 items, divided into multiple choice, single best choice and free text questions on personal practice as well as clinical scenarios (see supplement for entire list of questions).

Survey invitations were emailed to all ESNR members (*n* = 1662) and non-members who had expressed their interest in ESNR activities in the past (*n* = 6400), European national neuroradiological societies (the UK, the Netherlands, Belgium, Turkey), and distributed via professional social media channels (LinkedIn, Twitter, Facebook). The survey was open for 2 months from 1 May to 1 July 2017. To avoid duplicate bias, participants were instructed to supply institution details or confirm they were the only person answering from their centre.

A number of questions included the option ‘other’. If this was answered by < 5% of individuals, percentages are not quoted in the results.

The results of the survey were presented by the members of the working group at the 2017 annual meeting of the ESNR in Malmo in a dedicated session that was attended by around a hundred meeting participants. Matters arising in the plenary discussion of that session were considered when interpreting survey results and formulating conclusions and discussed further with the EAN representatives.

### Results

#### Demographic data and set-up of practice

**Fig. 1:**
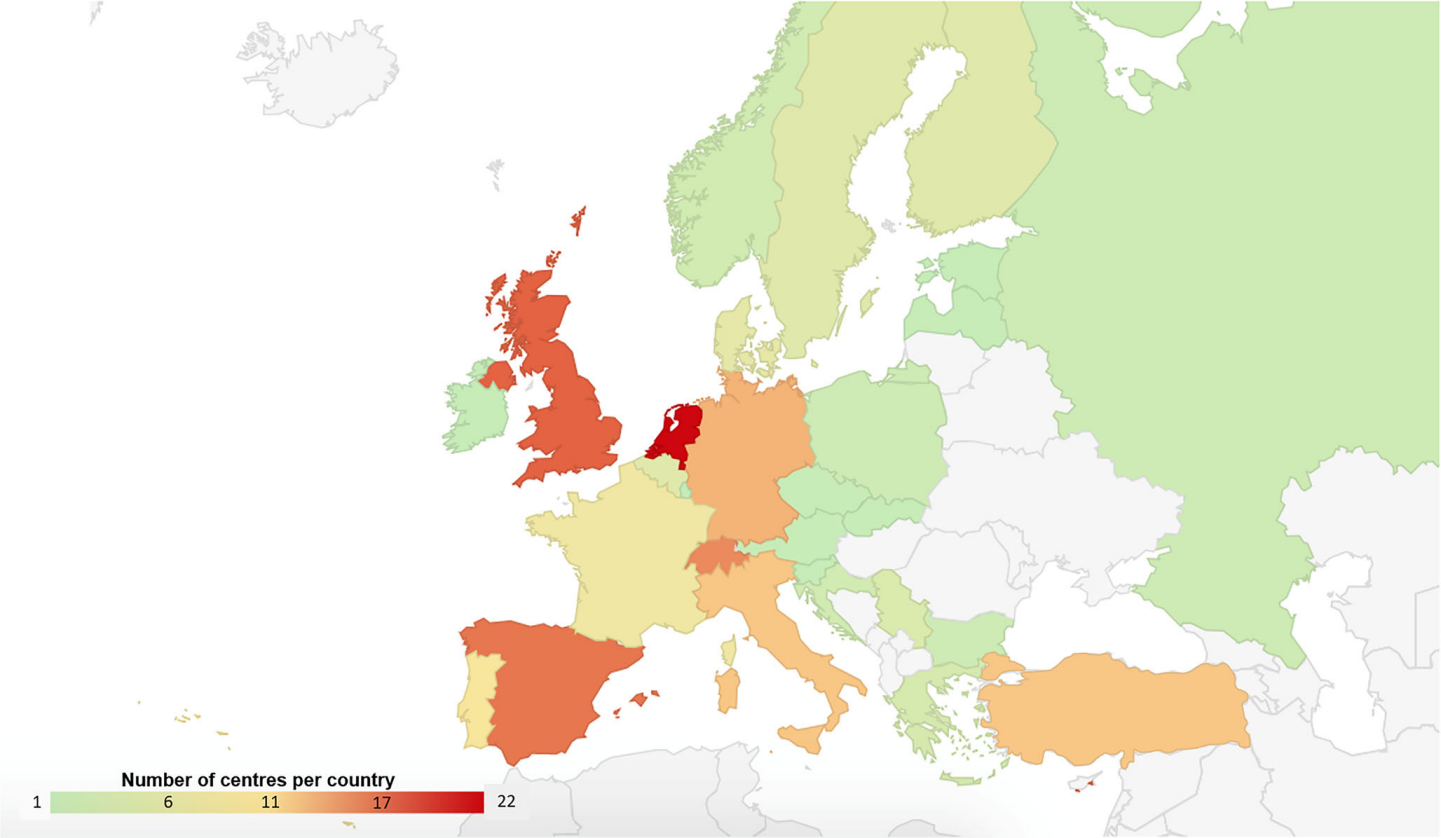
Institutional responses (number) per country. Countries with no responses are shaded grey

A total of 193 unique (non-duplicate) entries from European institutions were received from a total of 28 countries. Figure 1 shows a map of the distribution of responses per country. Countries with most participating institutions were the Netherlands (*n* = 22), the UK (19), Spain (18), Switzerland (17), Germany (15), Italy (14) and Turkey (14).

Among the 193 respondents, 75% were neuroradiologists, 12% worked as general radiologists and 11% were still in training. Of all respondents, 115 (59.6%) worked in academic hospitals, 66 (34.2%) in general hospitals, 27 in private practice (14.0%), 7 in memory clinics and 3 in other centres (note that multiple answers were possible to this question).

When we restricted the analysis to respondents who worked only in academia or in a non-academic setting (general hospital or private practice) (*n* = 173), 101 of those (58%) worked in academia and 72 (42%) in a non-academic setting. This sample excluded respondents (*n* = 20) who worked in both settings. We used this sample to stratify several of the analyses for an academic versus non-academic setting, to study whether important differences between both settings existed.

**Fig. 2:**
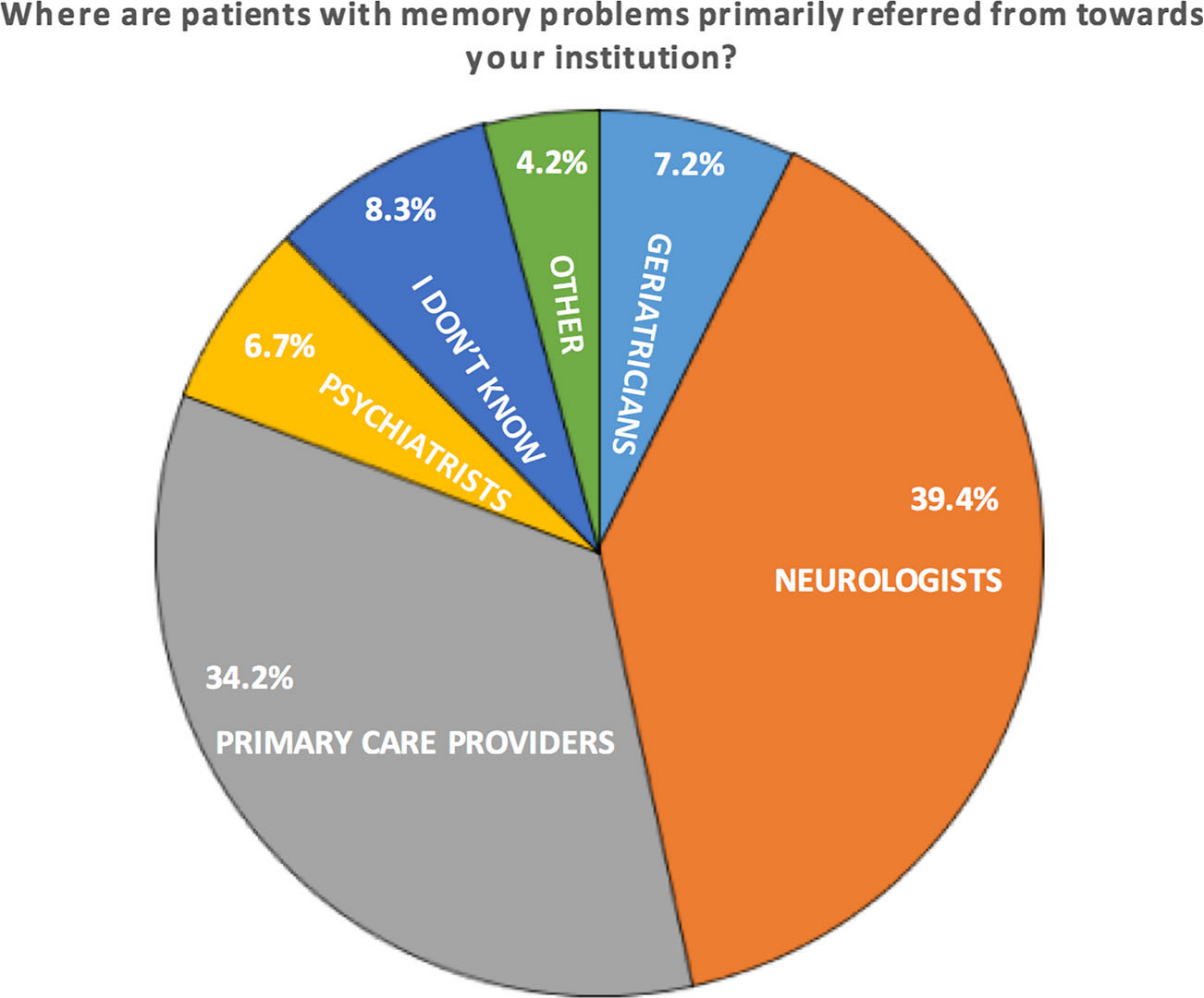
Referral categories

Specific memory clinics to evaluate patients were present in 121 (62.7%) of all institutions. This number was lower for general hospitals (48.6%) compared to academic institutions (71.3%). Patients were being referred for dementia evaluation primarily by neurologists (39.4%) and primary care providers (34.2%) (Fig. 2).

**Fig. 3:**
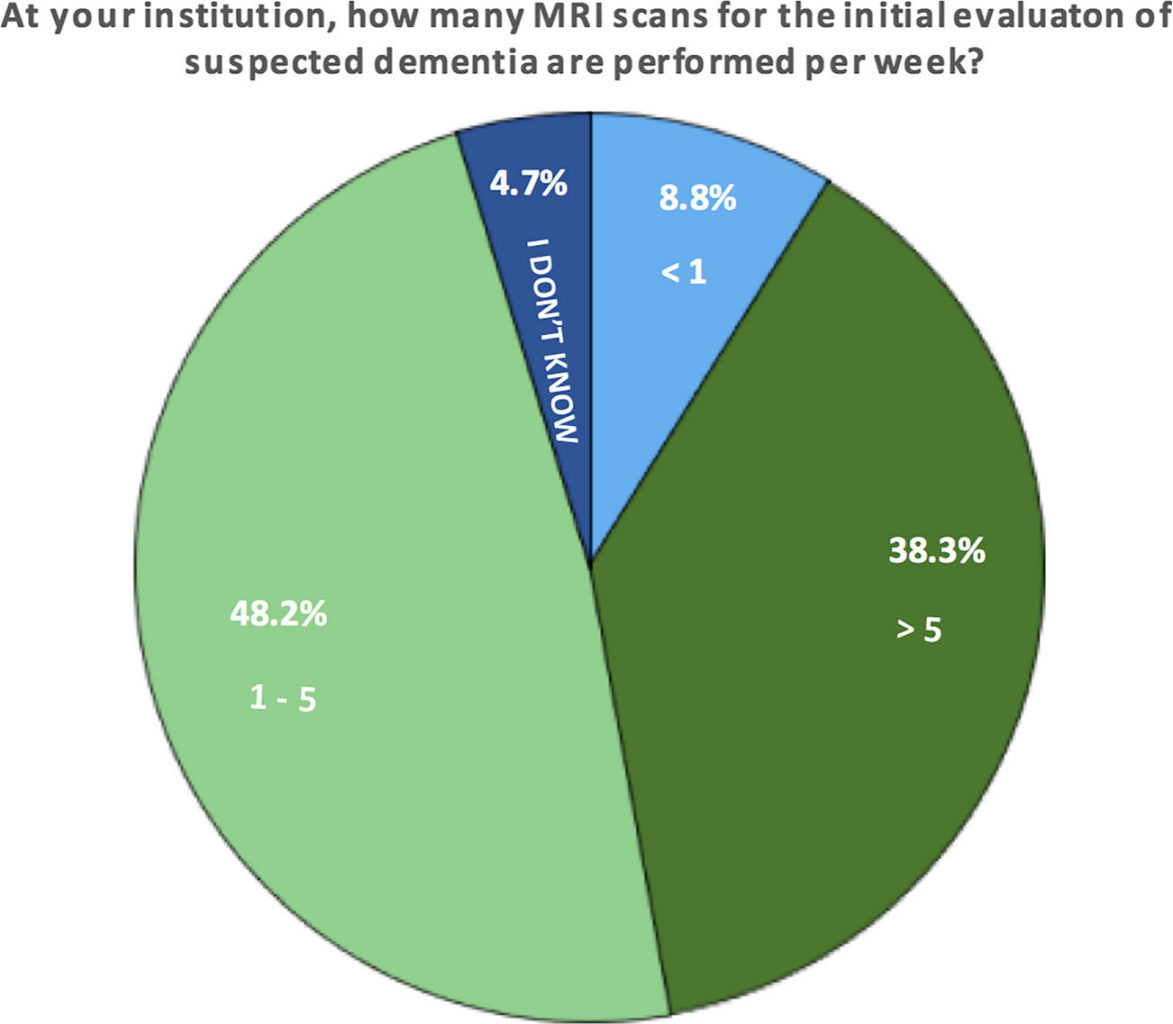
Caseload per centre for initial diagnosis of dementia

Of the 193 responding institutes, 38.3% performed more than five scans for the initial evaluation of dementia per week and 48.2% performed one to five scans per week (Fig. 3). These proportions did not differ significantly between academic centres and non-academic institutions, but scan numbers were slightly higher in academia (e.g. 43.6 vs. 34.7% performed > 5 scans per week).

#### Imaging modalities

Imaging modalities available in the 193 responding institutes were MRI (99.5%), CT (91.2%), single-photon emission computed tomography (SPECT) (either perfusion or dopamine transporter (DAT) scan) (47.7%), positron emission tomography (PET) (40.4%), PET-CT (43%) and PET-MRI (10.9%).

The primary imaging modality used for evaluation of patients suspected of dementia was CT in 49 (25.4%) of all institutions and MRI in 138 (71.5%). The remainder of institutions used both modalities equally (1.5%) or used MRI primarily in persons under the age of 65 years and CT in those who are older (1%). Non-academic institutions had a slightly stronger tendency towards MRI as primary modality than academic centres (73.6 versus 67.3%).

The 49 centres who used CT as primary modality did so mainly because of shorter wait time (73.5%) and lower costs (65.3%) and/or because CT was considered sufficient to answer the clinical questions (40.8%); only 8 (16.3%) of these 49 centres did not have access to MRI (multiple answers possible).

#### MR imaging protocol

Over 90% of all 193 institutions performed an MR imaging protocol consisting of the following structural sequences: T1-weighted (100%, of which 72.5% in 3D and 27.5% 2D acquisition), FLAIR (96.4%), T2-weighted (93.8%), T2*-weighted GRE or susceptibility-weighted imaging (SWI) (92.2%) and diffusion-weighted imaging (91.2%). Only 13% of institutions routinely performed T1-weighted imaging post-gadolinium-contrast-based agent administration. Coronal reformats for hippocampal assessment were made routinely in 80.9% of the 193 centres, usually (62.2%) by radiographers, and in 27.5% by radiologists themselves.

With respect to advanced imaging, only 48 (25%) of institutions performed any type of advanced imaging in routine diagnostic workup of dementia patients. Of these, the most frequent were ASL (*n* = 25), DTI (*n* = 23), spectroscopy (*n* = 15), resting-state fMRI (*n* = 12) or dynamic contrast-enhanced (DCE)/dynamic susceptibility contrast (DSC) perfusion (*n* = 7). Primary reasons mentioned not to perform advanced imaging were that clinicians do not request it (57.9%) because it is too time-intensive (59.7%), the sequences are not available (30.1%) or post-processing software is not available (23.5%—multiple answers possible).

More detailed reasons are shown in Supplementary Fig. 1.

#### Image interpretation: use of semi-quantitative rating scales

In 144 (74.6%) of all institutes, visual rating scales were used for scan interpretation. This proportion was slightly higher in non-academic (79.2%) than academic (69.3%) institutes. Reasons to *not* use rating scales (in 49 (25.4%) of institutes overall) were mainly that radiologists are not trained to use these (57.1%), that clinicians do not ask for this information (28.6%) or the use of these scales is considered too time-intensive (26.5%). Only 16.3% of the 49 non-users indicated that scales were ‘not useful’.

**Fig. 4:**
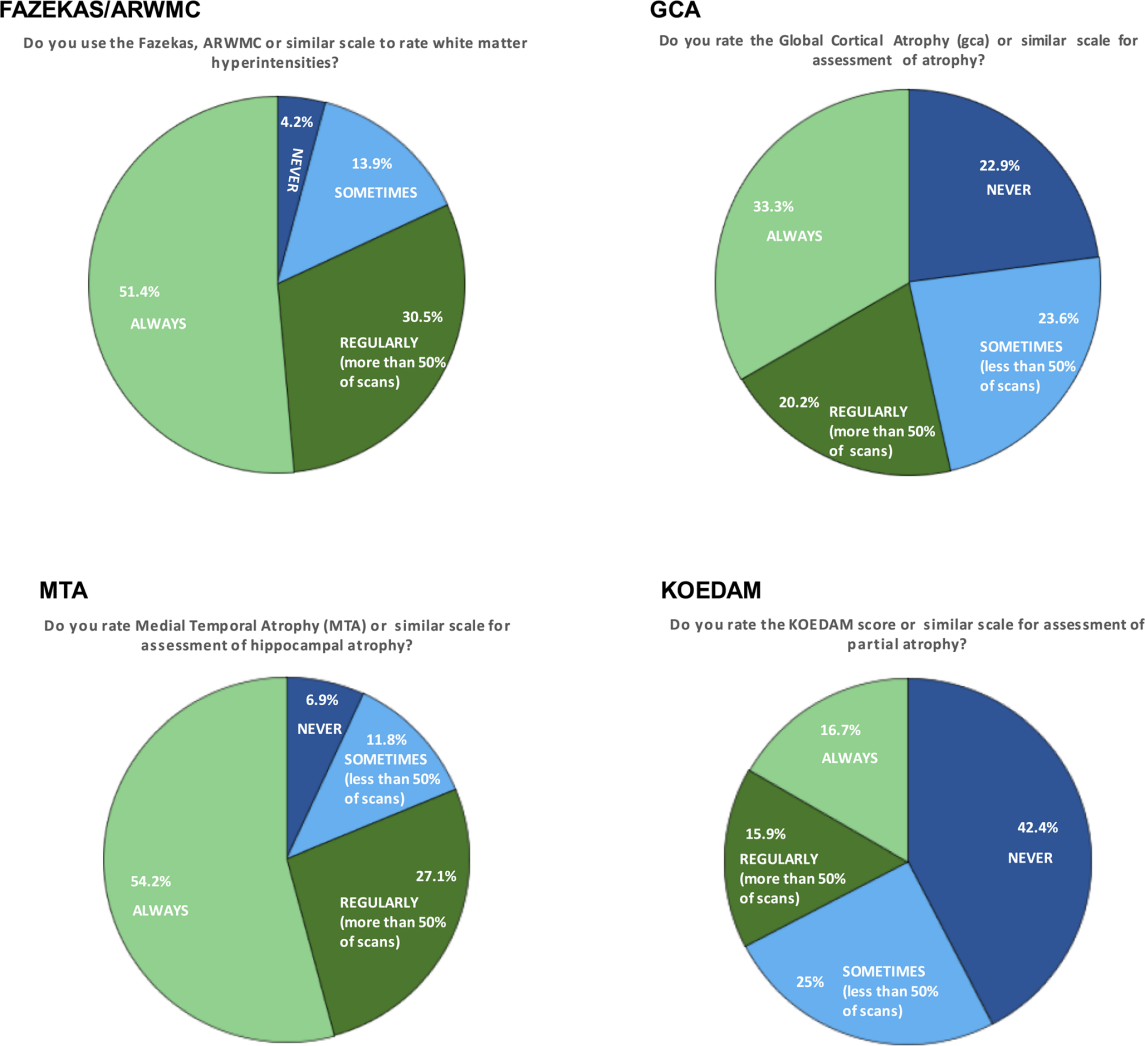
Use of visual rating scales. Pie charts show the frequency of use of each visual rating scale over all 193 institutes. Colours represent categories, and size of the coloured areas represents % of positive responses

Among the 144 institutes who used rating scales, Fig. 4 presents which types of scales were used and with what frequency. The survey questioned respondents whether they used each scale ‘always’ (in every scan), ‘regularly’ (in more than 50% of scans), ‘sometimes’ (in less than 50% of scans) or ‘never’. Most frequently used scales were white matter hyperintensity rating scales (used regularly or always in 81.9%) and the medial temporal atrophy (MTA) scale (used regularly or always in 81.3%). The global cortical atrophy (GCA) scale (53.4%) and the Koedam scale for posterior atrophy (32.6%) were less often applied. The relative frequency of use of visual rating scales did not differ between the academic and non-academic setting.

Confidence in use of these rating scales was again not different between academia and general hospitals, with 16.7% expressing high confidence and 50.7% reasonable confidence. Still, about a third of those using rating scales (*n* = 144 respondents) did not feel entirely confident (29.2%) or not confident at all (3.5%).

#### Use of quantitative information

Quantitative information, such as volumetric data, were used regularly in only 5.7% of the 193 centres, and in specific cases in 18.1% of institutes. Thus, in 147 (76.2%) institutes, volumetric information was never used. These proportions were similar for academic versus non-academic institutes.

Reasons *not* to perform quantitative evaluation were primarily the lack of access to software algorithms (70.1%) and the feeling that it would be too time-intensive (51.7%) (see more details in Supplementary Fig. 2).

Regarding the type of volumetric data used among those 46 institutes who did so regularly or in specific cases, hippocampal volume (in 39 (84.8%)) and total brain volume (in 30 (65.2%)) were most frequently measured, followed by white and grey matter volume (in 22 (47.8%)) and lobar volumes (in 17 (37%)). White matter hyperintensity volume was measured in only 21.7% of volumetric assessments.

Of the 46 centres that sometimes or regularly used volumetric data, 25 (54.3%) used normative reference data (e.g. percentile curves) to compare individual patients to. These reference data were derived from a large and heterogeneous range of data sources (varying from own samples, manufacturer provided or available open-access datasets).

Most of these 46 centres used the freely available FreeSurfer software (43.5%) for volumetric processing, followed at a distance by NeuroQuant (17.4%), AppMRI hippocampus volume analyser (15.2%) and Icometrix (4.3%).

#### Structured reporting

Structured reports were used in 54 (28.0%) of the 193 institutes, without differences between the academic and non-academic setting. The top reason not to use a structured report (in *n* = 139 institutes) was because radiologists do not find it useful (33.1%), followed by incompatibility with the speech recognition software (26.5%), time intensiveness (21.6%) and the fact that clinicians do not request structured reports (16.5%). In free text, 22 respondents (16%) mentioned that they are not used to it or do not have templates available.

#### Communication with clinicians

On the imaging request form, a detailed information regarding symptoms and the suspected diagnosis was provided by clinicians in only 57 institutes (29.5%), whereas only generic information ("query: dementia?") was provided in 130 (67.4%), and no information was provided in four (2.1%). These percentages did not differ substantially between academia (65% only generic information) and general institutions (73% only generic information).

In the conclusion of their radiological report, 60 (31.1%) of all respondents always indicated whether imaging findings support a diagnosis of dementia (and which subtype), whereas 108 (56%) only did so if the clinical diagnosis met the dementia criteria. Thirteen percent (13%) never included a statement on dementia in their conclusion. These numbers were equal for academia and general institutes.

In 19.1% of institutes (*n* = 37), cases suspected of dementia were always (4.1%) or regularly (more than 50% of new exams in 15%) discussed in multidisciplinary meetings, whereas this type of communication among specialists occurred ‘infrequently’ (less than 50% of patients) in 48.2% and ‘never’ in 32.6% of centres. If multidisciplinary meetings were held, these were primarily attended by neurologists (86.9%), radiologists (83.8%), neuropsychologists (41.5%) and geriatricians (41.5%). Psychiatrists were present in 30.8% of meetings; nuclear medicine physicians (15.4%), internists (9.2%), social workers (10%) and researchers (8.5%) less often so.

## Discussion on survey results and conclusions

### Generalisability of results

The ESNR survey on imaging in dementia had a reasonable number of responses (193 unique European centres), with a good geographical spread including Eastern-Europe. We should consider however that the low response rate (less than 5% of all invitations) will have led to selection bias, likely towards an overrepresentation of those who are very active and potentially have more expertise in this field. Indeed, the majority of respondents were subspecialty-trained neuroradiologists and working in academic centres, seeing roughly five scans per week for patients with dementia. Referrals tended to be from dedicated memory centres, though in non-academic centres, this was balanced with primary care referrals. We tried to take the academic overrepresentation into account by stratifying several results for academic versus non-academic centres and found that these did not differ, pointing towards generalisability and representativeness. Also, the response rate in this survey was very similar to an earlier pan-European survey conducted by the ESNR on glioma imaging [19]. Overall, we feel that the responses provide a reasonably realistic picture of neuroradiology practice for dementia in Europe.

### Imaging modality and protocol for dementia

The imaging modality of choice among the survey respondents clearly is MRI and, in those cases where CT was used, motivation varied and included reasons such as limited access to MRI or lack of clinical impact. Some respondents also reported that the information obtained with CT is sufficient, which is indeed justified for exclusion of other treatable causes of cognitive impairment, and to a certain extent also for atrophy patterns and more extensive vascular pathology when using multi-detector CT with coronal and sagittal reformats [20]. Yet, it should be noted that pathology such as microbleeds, areas of diffusion restriction, and more subtle patterns of vascular lesions (white matter pathology) and cortical atrophy are better evaluated on MRI.

There was very high convergence on the MR sequences used, with over 90% of respondents conducting the earlier suggested minimum set of MR imaging sequences for the evaluation of memory clinic patients (Box 1) [3, 4]. This indicates that MR imaging among the European centres that participated in the survey is already fairly homogeneous and meets current acceptable standards in the field.

**Box 1.** Proposed minimum MR imaging protocol for dementia imaging (adapted from [3, 4])

**Table:** 

**•** Sagittal 3D T1-weighted with sagittal and oblique/coronal reformats for evaluation of cortical atrophy and hippocampal atrophy. **•** Sagittal 2D axial FLAIR or 3D FLAIR with axial reformats for evaluation of white matter pathology and infarcts. **•** Axial T2-weighted for evaluation of non-neurodegenerative pathology and infratentorial or thalamic infarcts. **•** Axial T2*-weighted gradient echo or SWI for evaluation of microbleeds and macrohaemorrhage. **•** Axial DWI for evaluation of acute ischemia or diffusion restriction in Creutzfeld-Jakob disease. **•** Routine administration of contrast agent should not be applied.	

There was a limited use among survey respondents of advanced sequences, such as DTI and fMRI, in the majority due to lack of interest among clinicians or radiologists (> 70% of non-users), which is probably due to the lack of proven diagnostic performance or clinical impact as well as time intensiveness of the acquisition and post-processing in relation to lack of reimbursement. ASL was used most frequently, probably reflecting its potential to replace fluorodeoxyglucose (FDG)-PET [21] while being non-invasive. In addition to uncertain value of many of the advanced sequences, prolonged acquisition times and lack of tools/support to interpret the data were hurdles that were mentioned by the survey respondents. Inter-vendor differences in acquisition and analysis methods likely play a role as well in the low rates of implementation of advanced sequences.

### Interpretation of scans

Interpretation of MRI scans in patients with dementia was often (75% of all institutes) supported by the use of visual rating scales which were used even more often by non-academic centres. The most popular scales used were the MTA for hippocampal and medial temporal involvement (related to Alzheimer’s disease) and the Fazekas scale for white matter lesions of presumed cerebrovascular origin; both can be applied to CT as well [20]. Less frequently used were scales for GCA and posterior atrophy (as can be seen in younger-onset AD patients). Most respondents felt quite comfortable in applying the visual rating scales, although there clearly is a need for further training [15]. Although cut-off values for abnormality are age (and sex)-dependent [22], visual rating scales perform well in discriminating major dementing disorders [14] and have good inter-observer variability [23]. Box 2 summarises the most frequently used visual rating scales.

**Box 2.** Frequently used visual rating scales

**Table:** 

Routinely: **•** MTA scales to describe hippocampal atrophy **•** GCA to describe amount of global atrophy per lobe as well as any asymmetry **•** White matter hyperintensity scales (the Fazekas or ARWMC) for burden of vascular pathology Upon indication: **•** Posterior atrophy scale (the Koedam scale) (and other regional descriptors) (e.g. in suspected PCA and FTD)	

*GCA*, global cortical atrophy; *MTA*, medial temporal atrophy; *PCA*, posterior cortical atrophy; *FTD*, frontotemporal dementia; *ARWMC*, age-related white matter changes

### Use of volumetry in image interpretation: future development?

Quantitative methods such as MR volumetry are currently mainly used in research settings. As indicated in our survey, only 5.7% of centres performed volumetric analyses on a regular basis, despite the fact that for example voxel-based morphometry (VBM) was introduced already in 2000 [24], and more user-friendly automated segmentation algorithms such as FreeSurfer were introduced over 20 years ago.

Theoretical advances of implementation of volumetry are its potentially higher sensitivity to subtle changes, yielding an earlier detection of abnormality, an objective observer-independent assessment and higher sensitivity to change over time, e.g. in clinical trials. Yet, evidence from clinical practice supporting these advantages is still scarce and not unequivocal [25–29].

Main hurdles identified by the respondents were the limited availability of software, lack of time to use (offline) workstations and difficulties in the interpretation. The lack of transition of volumetric techniques into clinical routine is in our opinion furthermore mainly due to key issues that are common to the introduction of new biomarkers. These comprise lack of standardisation, lack of validation, concerns about specificity and the difficulty to translate research findings on a group level to the individual patient. For example, with respect to hippocampal volumetry, the rate of atrophy of AD patients is around 4.25% per year, yet only 1.25% per year in controls, i.e. 3% difference for a follow-up imaging of 1 year [30]. To be able to detect these small differences and apply these findings in a clinical setting would call for standardisation of image acquisition (field strength, vendor type, imaging parameters) as, for example, done in ADNI [31]. In addition, in a single time point setting, the use of reference populations or control groups representative for the population an individual patient was derived from is needed. Likewise, there are multiple software analysis tools and multiple data analysis parameters which may influence the obtained results.

### Communication between referring clinicians and radiologists

In most instances, referrals were not accompanied by detailed clinical information and only a minority of respondents participated regularly in multidisciplinary meetings where such information would have been available. This most likely reflects the previous practice of performing structural imaging in suspected dementia only with the aim to exclude a surgical lesion. The role of neuroimaging in dementia has however changed importantly with imaging being incorporated in research and diagnostic criteria of several dementia subtypes [5, 7]. This stresses the need for close communication between referring clinicians and radiologists to obtain the most valuable information with respect to differential diagnosis.

In contrast, a general lack of clinical information may also drive a radiologist to scrutinise the images in a systematic fashion (rather than to look for a confirmatory finding) and may be more objective than being potentially misled by the clinical information. For example, in the clinical diagnosis of AD, about a third of cases cannot be confirmed and finding some degree of hippocampal atrophy could be misinterpreted when other diagnoses are not systematically considered (e.g. FTD).

Participation of multidisciplinary team (MDT) meetings was variable, although if these happened, radiologists were frequently included. Recognising that MDT meetings require time for preparation and attendance, we strongly recommend radiologists to participate, as it will allow them to address specific clinical information that may have arisen after the request form was issued or the patient was examined in more detail and the radiological findings can now be interpreted in light of additional laboratory (e.g. CSF) findings. Although numbers are not known for dementia MDT meetings, studies from the field of oncology show that radiological input during MDT meetings results in direct influence on patient management in up to 20% of discussed cases [32, 33]. MDT meetings are also important to generate feedback on the quality of the radiology reports and provide a mechanism to verify the accuracy of the conclusions and the appropriateness of the wording of reports. Finally, they provide an excellent training environment for younger colleagues.

### Structured analysis and reporting

Literature points towards the fact that semi-structured reporting may help to more systematically evaluate scans, remove ambiguity, lead to more clarity towards clinicians and facilitate research [34]. Yet in the majority of responding institutes (72% out of 193), radiologists did not apply structured reporting in dementia imaging, with as most common reasons not being supported by dictation software, being time-consuming or not helpful. Despite this, the frequent use of visual rating scales indicates there at least is a semi-structured approach to the interpretation of images. In fact, use of a standard set of visual rating scales (see Box 2) and measurements is a way to structure the analysis path and build the report systematically. In addition, the use of a common language may further be facilitated by the use of visual rating scales, which are more objective than subjective terms such as ‘mild’, ‘appreciable’ or ‘significant’. Interestingly, the use of semi-quantitative scales seemed to be adopted more widely in non-academic centres for reasons not further explored in the survey.

Finally, a report should always be placed in context of the provided information and question. The conclusion can mention whether there is neurodegeneration (and vascular) pathology beyond expectation for ageing, but, unless specifically asked to confirm a specific diagnosis, should only provide a possible differential diagnosis, to avoid anxiety and confusion among referrers and patients. This caveat becomes increasingly important as patients are getting direct access to reports through electronic health records and may even read the report before seeing the clinician.

Box 3 indicates a suggested structure for reporting in dementia imaging [3, 4].

**Box 3.** Suggested structure for reporting in dementia imaging (adapted from [3, 4])

**Table:** 

• Mention the scan protocol used and whether there are historical scans for comparison. • Exclude mass lesion or other (non-)surgical disorders that explain cognitive impairment other than neurodegeneration. • Describe vascular pathology › Include the Fazekas (or ARWMC) score for WMH. › Specify whether there are infarcts or microbleeds, including number and location. • Atrophy pattern › Provide MTA score. › GCA score, describe any lobar preference and asymmetry. › Describe infratentorial atrophy (mesencephalon, pons, cerebellum). Other relevant pathology › Mention any diffusion abnormalities. › Mention presence of hydrocephalus (communicating or non-communicating). • Conclusion—take age (and symptoms) into account. › Normal, vascular, neurodegenerative, mixed pathology. › Suggest DD for neurodegeneration (only if referral states that patient has dementia).	

### Conclusion and future outlook

Given the survey findings, we conclude that current practice in dementia imaging is fairly homogeneous across Europe, in terms of image acquisition and image interpretation. With respect to protocol set-up, relatively easy adaptation towards the best clinical practice recommendations can be expected, although some variations throughout Europe are likely to remain, depending on reimbursement strategies, practical and logistical setups and availability of scanning facilities. Several hurdles have been identified through the survey that may help progress the field further. These mainly lie in the areas of training on the use of visual rating scales, translation of research findings (advanced imaging) to a clinical setting, implementation of volumetric assessment, structured reporting and communication.

## Electronic supplementary material

ESM 1 (DOCX 118 kb)
