# Supplementary material for: Dementia imaging in clinical practice: a European-wide survey of 193 centres and conclusions by the ESNR working group
Source: Neuroradiology. 2019 Mar 9;61(6):633–42. doi: 10.1007/s00234-019-02188-y (PMC6511357; doi:10.1007/s00234-019-02188-y)

**Supplementary figure 1.** Reasons not to perform advanced imaging in dementia.

The figure shows the main reasons mentioned among the 145 institutes that did not perform any type of advanced imaging. Blue bars represent percentages for how often each reason not to perform advanced imaging was mentioned. Multiple reasons could be mentioned, so percentages do not add up to 100%.


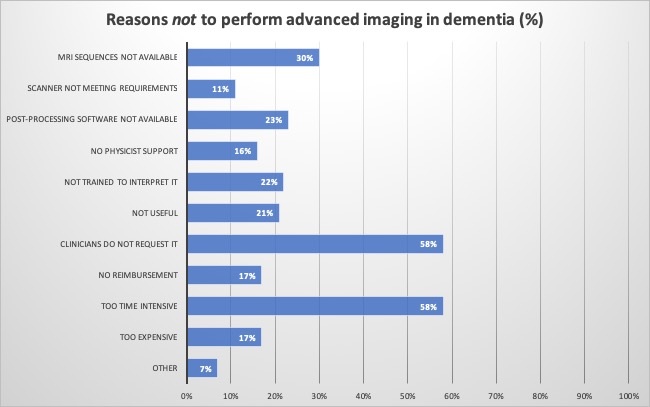


**Supplementary figure 2.** Reasons not to perform quantitative evaluation.

The figure shows the main reasons mentioned among the 147 institutes that did not perform quantitative evaluation. Blue bars represent percentages for how often each reason not to perform quantitative evaluation was mentioned. Multiple reasons could be mentioned, so percentages do not add up to 100%.


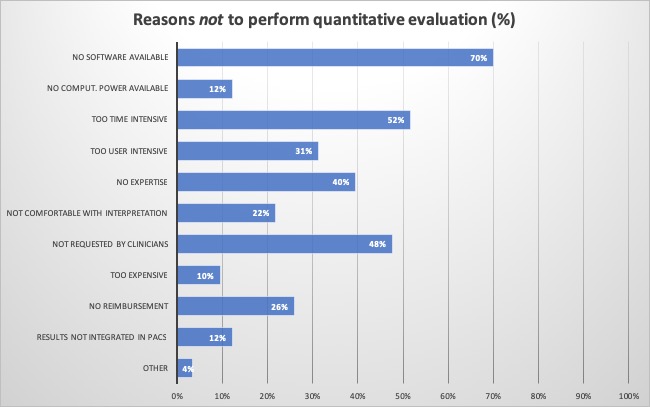

Supplement: Supplementary file 1 — (DOCX 118 kb) [file 234_2019_2188_MOESM1_ESM.docx]
